# Crystal and mol­ecular structure of 2-methyl-1,4-phenyl­ene bis­(3,5-di­bromo­benzoate)

**DOI:** 10.1107/S2056989024006820

**Published:** 2024-07-15

**Authors:** Nathan J. Weeks, Moira K. Lauer, Gary J. Balaich, Scott T. Iacono

**Affiliations:** a Department of Chemistry & Chemistry Research Center, USAF Academy, Colorado Springs, CO 80840, USA; Vienna University of Technology, Austria

**Keywords:** crystal structure, inverse vulcanization (InV), RASP, Steglich esterification, di­thio­carbamate (DTC) catalyst

## Abstract

The aryl diester compound, 2-methyl-1,4-phenyl­ene bis­(3,5-di­bromo­benzoate), C_21_H_12_Br_4_O_4_, was synthesized by esterification of methyl hydro­quinone with 3,5-di­bromo­benzoic acid. A crystalline sample was obtained by cooling a sample of the melt (m.p. = 502 K/DSC) to room temperature. The mol­ecular structure consists of a central benzene ring with *anti*-3,5-di­bromo­benzoate groups symmetrically attached at the 1 and 4 positions and a methyl group attached at the 2 position of the central ring. In the crystal structure (space group *P*), mol­ecules of the title aryl diester are located on inversion centers imposing disorder of the methyl group and H atom across the central benzene ring. The crystal structure is consolidated by a network of C—H⋯Br hydrogen bonds in addition to weaker and offset π–π inter­actions involving the central benzene rings as well as the rings of the attached 3,5-di­bromo­benzoate groups.

## Abstract 2

Mol­ecules of the aryl diester, 2-methyl-1,4-phenyl­ene bis­(3,5-di­bromo­benzoate), crystallized out from the melt (m.p. = 502 K/DSC). The crystal structure consists of a C—H⋯Br hydrogen-bonded network and weaker, offset π–π inter­actions.

## 1. Chemical context

Inverse vulcanization (InV) polymerization is an important solvent-less process for the synthesis of elastomeric materials from elemental sulfur and thermally stable organic co-monomers, both of which are often found as waste products of the chemical industry (Chung *et al.*, 2013 ▸; Karunarathna *et al.*, 2020 ▸). Recently, aryl halide co-monomers, including the title aryl diester, were investigated for un-catalyzed InV chemistry, and shown to react *via* a radical aryl sulfur polymerization (RASP) mechanism at temperatures > 493 K (Karunarathna *et al.*, 2020 ▸; Thio­unn *et al.*, 2020 ▸). An advantage of the title aryl diester as a co-monomer for InV reactions is reflected by its conjugated aromaticity and attendant exceptional thermal stability (*T*_d_ = 563 K/TGA). Further, a more recent study (Lauer *et al.*, 2024 ▸) demonstrated that successful InV reactions could be carried out at temperatures as low as 463 K, using the title aryl diester co-monomer in conjunction with a di­thio­carbamate (DTC) catalyst. The catalyzed reaction data were significant because they provided evidence for the possible involvement of anionic sulfur inter­mediates and expanded the possible scope of the InV reactions to more thermally sensitive co-monomers (Lauer *et al.*, 2024 ▸).
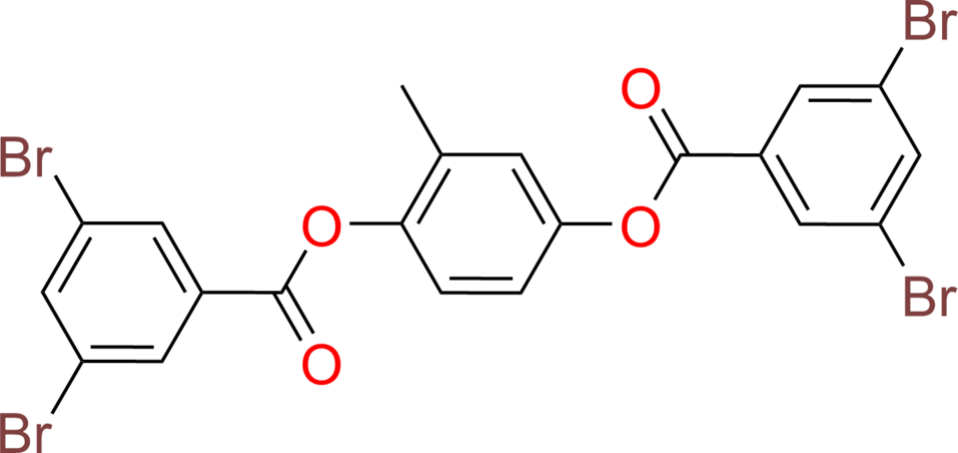


## 2. Structural commentary

**Figure 1:**
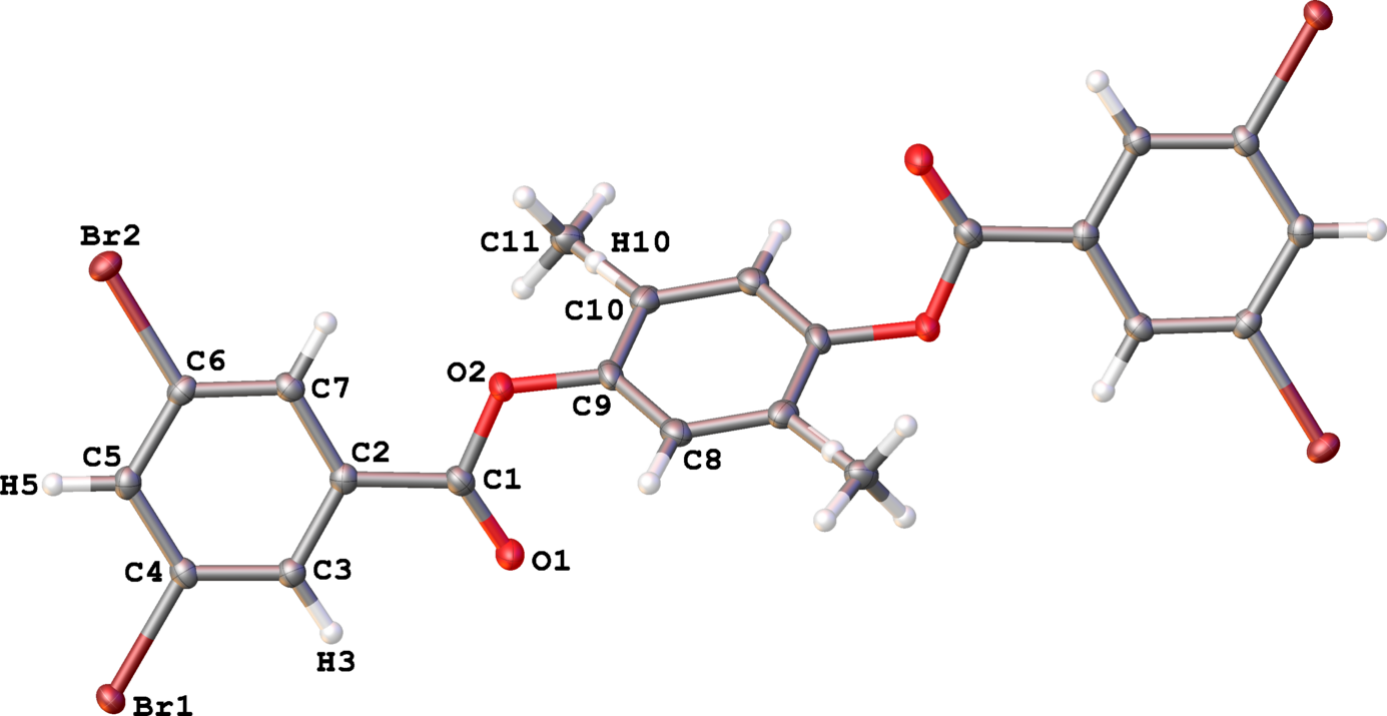
Mol­ecular structure of 2-methyl-1,4-phenyl­ene bis­(3,5-di­bromo­benzoate), depicting the *anti*-position of the 3,5-di­bromo­benzoate end groups. The methyl group is shown in both positions, disordered across the central benzene ring in space group *P*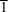. Displacement ellipsoids are shown at the 50% probability level; non-labeled atoms are generated by symmetry operation 1 − *x*, 2 − *y*, −*z*.

The aryl diester compound, 2-methyl-1,4-phenyl­ene bis­(3,5-di­bromo­benzoate), crystallizes in the space group *P*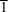 with one half mol­ecule per asymmetric unit. Mol­ecules lie on crystallographic inversion centers that impose disorder of the methyl group (C11H_3_) and an H atom (H10) across the central benzene ring (Fig. 1 ▸). The two 3,5-di­bromo­benzoate end groups are attached to the central benzene ring in an *anti* fashion, with the planes of the 3,5-di­bromo­benzoate rings inclined at a dihedral angle of 54.53 (9)° with respect to the plane of the central benzene ring (Fig. 1 ▸). The ester groups are nearly co-planar with their conjugated 3,5-di­bromo­phenyl rings, making a dihedral angle of only 8.21 (11)°, but inclined at a dihedral angle of 62.58 (10)° with respect to the central benzene ring (Fig. 1 ▸). This compares well to the structure of the related 1,4-phenyl­ene dibenzoate, with the end group rings and ester groups tipped with respect to the central 1,4-benzene ring at dihedral angles of 55.29 (8) and 60.31 (9)°, respectively, and the ester groups with their conjugated end group rings tipped at only a small dihedral angle of 5.94 (8)° (Ganaie *et al.*, 2016 ▸).

## 3. Supra­molecular features

**Figure 2:**
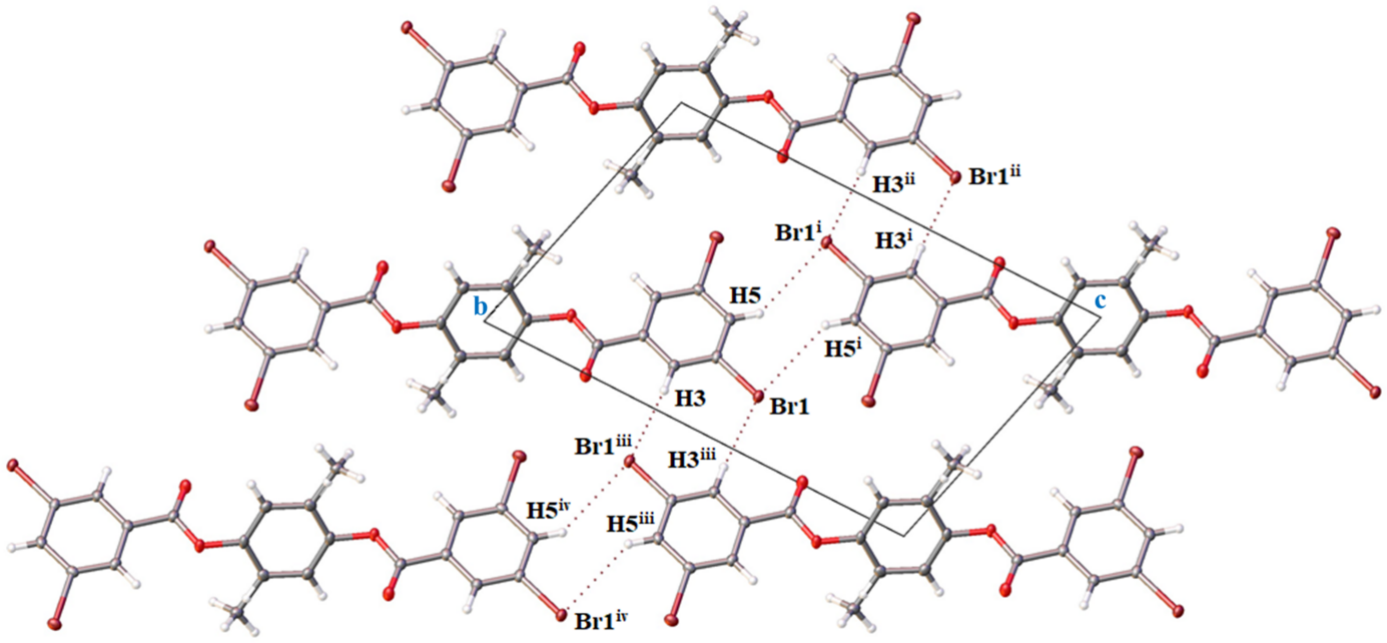
Hydrogen-bonding motif for 2-methyl-1,4-phenyl­ene bis­(3,5-di­bromo­benzoate), depicting complementary end-to-end [C5—H5⋯Br1, 3.12 (2) Å] and side-to-side [C3—H3⋯Br1, 3.02 (1) Å] C—H⋯Br inter­actions, in a view down [100]. Displacement ellipsoids are shown at the 50% probability level. [Symmetry codes: (i) 1 − *x*, 1 − *y*, 1 − *z*; (ii) −1 + *x*, −1 + *y*, *z*; (iii) 2 − *x*, 2 − *y*, 1 − *z*; (iv) 1 + *x*, 1 + *y*, *z*.]

**Figure 3:**
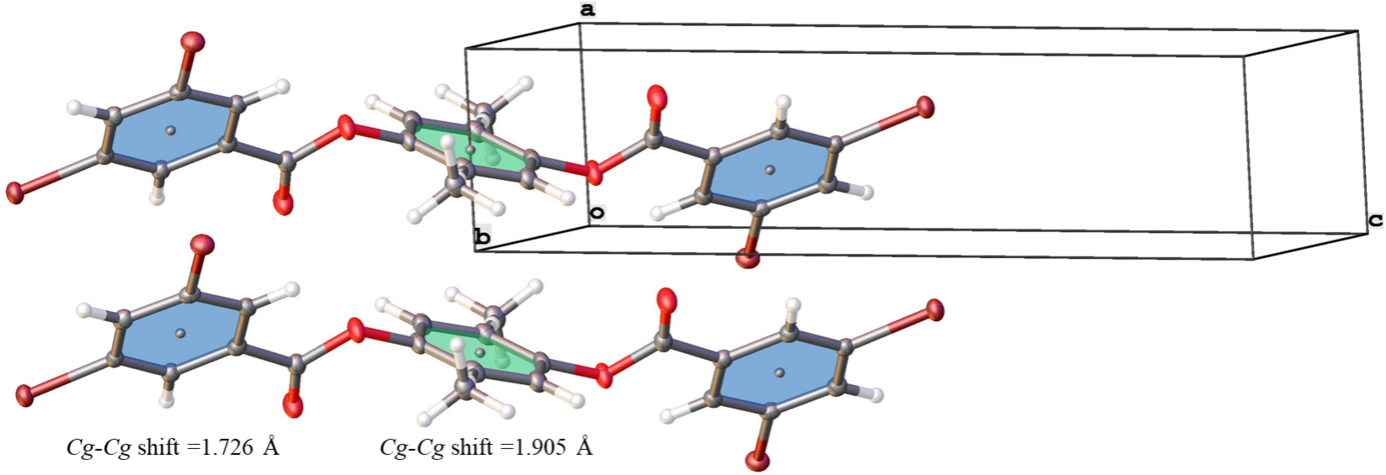
Unit-cell overlay and depiction of the π–π inter­actions along [100] in the crystal structure of 2-methyl-1,4-phenyl­ene bis­(3,5-di­bromo­benzoate), giving parallel but slipped central 1,4-benzene rings (shaded green) and end group 3,5-di­bromo­phenyl rings (shaded blue). The centroids of the central benzene rings lie on inversion centers, giving equal *Cg⋯*Cg** distances that correspond to the *a* lattice parameter [3.8875 (1) Å] with ring centroids shifted by 1.905 Å (central benzene rings) and 1.726 Å (3,5-di­bromo­phenyl rings). Displacement ellipsoids are shown at the 50% probability level.

Inter­molecular contacts of the title aryl diester involve hydrogen-bonding and weaker ring π–π inter­actions. Complementary end-to-end hydrogen bonding C5—H5⋯Br1 [3.12 (2) Å, Table 1 ▸] between the 3,5-di­bromo­phenyl groups forms chains of aryl diester mol­ecules that run parallel to [011] (Fig. 2 ▸). The planes between the 3,5-di­bromo­phenyl rings on adjacent mol­ecules in the chains are offset, giving a stair-step pattern of aryl diester mol­ecular links in the chains (Fig. 2 ▸). Complementary C3—H3⋯Br1 inter­actions [3.02 (1) Å, Table 1 ▸] extend along [100] and result in shorter side-to-side hydrogen bonding that cross-links the end-to-end chains forming a tri-periodic network (Fig. 2 ▸). This arrangement places the Br2 and H7 atoms in positions that point towards the central benzene rings of adjacent mol­ecules and that are free of C—H⋯Br hydrogen-bonding inter­actions (Fig. 2 ▸). The network of C—H⋯Br hydrogen bonds between Br1 and H3/H5 leaves a side-to-side packing of mol­ecules along [100] with all rings on adjacent mol­ecules oriented parallel (Fig. 3 ▸). Weak π–π inter­actions are evident between these parallel rings with centroid-to-centroid (*Cg⋯*Cg**) distances of 3.8875 (1) Å, but with their centroids shifted by 1.726 Å (3,5-di­bromo­phenyl rings) and 1.905 Å (central benzene rings). In the crystal structure of the related 1,4-phenyl­ene dibenzoate, three C—H⋯π inter­actions and one displaced π–π inter­action between the peripheral rings [*Cg⋯*Cg** distance = 3.9590 (10) Å] were noted (Ganaie *et al.*, 2016 ▸). The presence of Br and the attendant network of stronger C—H⋯Br hydrogen bonds in the title aryl diester structure precludes C—H⋯π inter­actions, resulting in only displaced and weak π–π inter­actions between parallel rings.

## 4. Database survey

Five structurally related aryl ester compounds were found in the Cambridge Structure Database [CSD; web inter­face (CCDC 2017); Groom *et al.*, 2016 ▸]. Of the three aryl diesters reported, two contain a central 1,4-benzene ring bound at both positions to either an unsubstituted benzoate group [CSD entry NADMUD (deposition number 1407716); Ganaie *et al.*, 2016 ▸] or to a *p*-tolyl benzoate group [CSD entry TAJDEN (deposition number 1265699); Ciajolo *et al.*, 1991 ▸], and one contains a central 9,10-anthra­hydro­quinone ring bound at both positions to an unsubstituted benzoate group [CSD entry ANTHQB (deposition number 1103109); Iball & Mackay, 1962 ▸]. The remaining two hits are the monoesters, 4-bromo­phenyl benzoate [CSD entry QIXNER (deposition number 684565); Gowda *et al.*, 2008 ▸] and 4-meth­oxy­phenyl benzoate [CSD entry TIGVUB (deposition number 657773); Gowda *et al.*, 2007 ▸]. Hydrogen bonding was not observed in the crystal structure of 4-bromo­phenyl benzoate (Gowda *et al.*, 2008 ▸).

## 5. Synthesis and crystallization

The synthesis of 2-methyl-1,4-phenyl­ene bis­(3,5-di­bromo­benzoate) was carried out using a modified Steglich esterification procedure and was previously published (Lauer *et al.*, 2024 ▸; Jordan *et al.*, 2021 ▸). M.p. = 502 K/DSC, *T*_d_ = 563 K/TGA). A crystalline sample was obtained by melting a sample of a white powder of 2-methyl-1,4-phenyl­ene bis­(3,5-di­bromo­benzoate) in a glass vial on a hot plate. The melt was allowed to cool to room temperature, forming a crystalline solid. Crystals suitable for single crystal X-ray diffraction were obtained by cutting into the solidified crystalline melt sample.

## 6. Refinement

Crystal data, data collection and structure refinement details are summarized in Table 2 ▸. All hydrogen atoms, except H3, H5 and H10, were placed using a riding model with their positions constrained relative to their parent C atom using the appropriate HFIX command in *SHELXL* (Sheldrick, 2015*b* ▸). Hydrogen atoms involved in C—H⋯Br hydrogen-bonding, H3 and H5, as well as H10 were placed from the electron-density map, and their C—H distances restrained (DFIX, C—H range 0.94–0.95 Å) at 0.95 Å with *U*_iso_(H) = 1.2*U*_eq_(C). Electron density corresponding to the disordered methyl group (C11) and H atom (H10) positions was obvious in the electron-density map. The occupancies of disordered atoms, H10 and C11, were set to 0.5, and H atoms attached to C11 (H11*A*, H11*B*, and H11*C*) were placed using a riding model (HFIX 137).

## Supplementary Material

Crystal structure: contains datablock(s) I. DOI: 10.1107/S2056989024006820/wm5727sup1.cif

Structure factors: contains datablock(s) I. DOI: 10.1107/S2056989024006820/wm5727Isup2.hkl

Supporting information file. DOI: 10.1107/S2056989024006820/wm5727Isup6.cdx

DSC thermogram. DOI: 10.1107/S2056989024006820/wm5727sup4.tif

Supporting information file. DOI: 10.1107/S2056989024006820/wm5727sup5.tif

Supporting information file. DOI: 10.1107/S2056989024006820/wm5727Isup6.cml

CCDC reference: 2363671

Additional supporting information:  crystallographic information; 3D view; checkCIF report

## Figures and Tables

**Table 1:** Hydrogen-bond geometry (Å, °)

*D*—H⋯*A*	*D*—H	H⋯*A*	*D*⋯*A*	*D*—H⋯*A*
C5—H5⋯Br1^i^	0.95 (1)	3.12 (2)	3.893 (2)	139 (2)
C3—H3⋯Br1^ii^	0.95 (1)	3.02 (1)	3.956 (2)	172 (2)

Symmetry codes: (i) 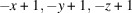; (ii) 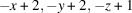.

**Table 2:** Experimental details

Crystal data	
Chemical formula	C_21_H_12_Br_4_O_4_
*M*_r_	647.95
Crystal system, space group	Triclinic, *P* 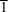
Temperature (K)	100
*a*, *b*, *c* (Å)	3.8875 (1), 9.3118 (2), 14.7772 (3)
α, β, γ (°)	104.228 (2), 93.211 (2), 98.219 (2)
*V* (Å^3^)	510.87 (2)
*Z*	1
Radiation type	Cu *K*α
μ (mm^−1^)	9.85
Crystal size (mm)	0.13 × 0.06 × 0.01

Data collection	
Diffractometer	XtaLAB Synergy, Dualflex, HyPix3000
Absorption correction	Gaussian (*CrysAlis PRO*; Rigaku OD, 2024 ▸)
*T*_min_, *T*_max_	0.543, 0.998
No. of measured, independent and observed [*I* > 2σ(*I*)] reflections	9500, 1892, 1848
*R*_int_	0.022
(sin θ/λ)_max_ (Å^−1^)	0.605

Refinement	
*R*[*F*^2^ > 2σ(*F*^2^)], *wR*(*F*^2^), *S*	0.019, 0.048, 1.11
No. of reflections	1892
No. of parameters	147
No. of restraints	3
H-atom treatment	H atoms treated by a mixture of independent and constrained refinement
Δρ_max_, Δρ_min_ (e Å^−3^)	0.35, −0.49

Computer programs: *CrysAlis PRO* (Rigaku OD, 2024 ▸), *SHELXT* (Sheldrick, 2015*a* ▸), *SHELXL* (Sheldrick, 2015*b* ▸), and *OLEX2* (Dolomanov *et al.*, 2009 ▸).

## supplementary crystallographic information

### 2-Methyl-1,4-phenylene bis(3,5-dibromobenzoate) Crystal data

**Table:**

C_21_H_12_Br_4_O_4_	*Z* = 1
*M**_r_* = 647.95	*F*(000) = 310
Triclinic, *P*1	*D*_x_ = 2.106 Mg m^−^^3^
*a* = 3.8875 (1) Å	Cu *K*α radiation, λ = 1.54184 Å
*b* = 9.3118 (2) Å	Cell parameters from 8434 reflections
*c* = 14.7772 (3) Å	θ = 3.1–68.8°
α = 104.228 (2)°	µ = 9.85 mm^−^^1^
β = 93.211 (2)°	*T* = 100 K
γ = 98.219 (2)°	Plate, clear colourless
*V* = 510.87 (2) Å^3^	0.13 × 0.06 × 0.01 mm

### 2-Methyl-1,4-phenylene bis(3,5-dibromobenzoate) Data collection

**Table:**

XtaLAB Synergy, Dualflex, HyPix3000 diffractometer	1892 independent reflections
Radiation source: micro-focus sealed X-ray tube, PhotonJet (Cu) X-ray Source	1848 reflections with *I* > 2σ(*I*)
Mirror monochromator	*R*_int_ = 0.022
Detector resolution: 10.0000 pixels mm^-1^	θ_max_ = 68.9°, θ_min_ = 3.1°
ω scans	*h* = −4→4
Absorption correction: gaussian (CrysAlisPro; Rigaku OD, 2024)	*k* = −11→11
*T*_min_ = 0.543, *T*_max_ = 0.998	*l* = −17→17
9500 measured reflections	

### 2-Methyl-1,4-phenylene bis(3,5-dibromobenzoate) Refinement

**Table:**

Refinement on *F*^2^	Hydrogen site location: mixed
Least-squares matrix: full	H atoms treated by a mixture of independent and constrained refinement
*R*[*F*^2^ > 2σ(*F*^2^)] = 0.019	*w* = 1/[σ^2^(*F*_o_^2^) + (0.021*P*)^2^ + 0.7349*P*] where *P* = (*F*_o_^2^ + 2*F*_c_^2^)/3
*wR*(*F*^2^) = 0.048	(Δ/σ)_max_ = 0.001
*S* = 1.11	Δρ_max_ = 0.35 e Å^−^^3^
1892 reflections	Δρ_min_ = −0.49 e Å^−^^3^
147 parameters	Extinction correction: SHELXL (Sheldrick, 2015*b*), Fc^*^=kFc[1+0.001xFc^2^λ^3^/sin(2θ)]^-1/4^
3 restraints	Extinction coefficient: 0.00125 (19)
Primary atom site location: dual	

### 2-Methyl-1,4-phenylene bis(3,5-dibromobenzoate) Special details

**Table:**

Geometry. All esds (except the esd in the dihedral angle between two l.s. planes) are estimated using the full covariance matrix. The cell esds are taken into account individually in the estimation of esds in distances, angles and torsion angles; correlations between esds in cell parameters are only used when they are defined by crystal symmetry. An approximate (isotropic) treatment of cell esds is used for estimating esds involving l.s. planes.

### 2-Methyl-1,4-phenylene bis(3,5-dibromobenzoate) Fractional atomic coordinates and isotropic or equivalent isotropic displacement parameters (Å^2^)

**Table:**

	*x*	*y*	*z*	*U*_iso_*/*U*_eq_	Occ. (<1)
Br1	0.70739 (6)	0.79521 (3)	0.55753 (2)	0.01735 (10)	
Br2	−0.10300 (6)	0.36527 (3)	0.25588 (2)	0.01878 (10)	
O1	0.7673 (5)	1.00485 (19)	0.24435 (12)	0.0222 (4)	
O2	0.3733 (4)	0.84273 (18)	0.13414 (11)	0.0195 (4)	
C1	0.5529 (6)	0.8933 (3)	0.22059 (16)	0.0171 (5)	
C2	0.4527 (6)	0.7893 (3)	0.28060 (16)	0.0154 (5)	
C3	0.5910 (6)	0.8343 (3)	0.37435 (16)	0.0152 (5)	
C4	0.5110 (6)	0.7388 (3)	0.43104 (16)	0.0151 (4)	
C5	0.3006 (6)	0.6000 (3)	0.39750 (17)	0.0165 (5)	
C6	0.1688 (6)	0.5579 (3)	0.30378 (17)	0.0154 (5)	
C7	0.2403 (6)	0.6506 (3)	0.24487 (16)	0.0156 (5)	
H7	0.146265	0.620309	0.181107	0.019*	
C8	0.3712 (6)	1.0708 (3)	0.08300 (17)	0.0185 (5)	
H8	0.282846	1.117184	0.139659	0.022*	
C9	0.4468 (6)	0.9264 (3)	0.06787 (16)	0.0176 (5)	
C10	0.5725 (6)	0.8531 (3)	−0.01344 (17)	0.0186 (5)	
H10	0.611 (18)	0.754 (3)	−0.019 (5)	0.022*	0.5
C11	0.6348 (14)	0.6943 (5)	−0.0242 (4)	0.0197 (10)	0.5
H11A	0.743277	0.660759	−0.082337	0.030*	0.5
H11B	0.411636	0.628836	−0.026516	0.030*	0.5
H11C	0.789947	0.689846	0.029312	0.030*	0.5
H5	0.249 (7)	0.535 (3)	0.4374 (17)	0.024*	
H3	0.742 (6)	0.9274 (18)	0.3950 (19)	0.024*	

### 2-Methyl-1,4-phenylene bis(3,5-dibromobenzoate) Atomic displacement parameters (Å^2^)

**Table:**

	*U*^11^	*U*^22^	*U*^33^	*U*^12^	*U*^13^	*U*^23^
Br1	0.01993 (15)	0.01909 (14)	0.01311 (14)	0.00223 (10)	−0.00038 (9)	0.00523 (9)
Br2	0.01717 (15)	0.01470 (14)	0.02246 (15)	−0.00116 (9)	−0.00003 (10)	0.00357 (10)
O1	0.0260 (9)	0.0214 (9)	0.0175 (8)	−0.0055 (7)	−0.0024 (7)	0.0080 (7)
O2	0.0247 (9)	0.0187 (8)	0.0141 (8)	−0.0039 (7)	−0.0037 (7)	0.0077 (7)
C1	0.0191 (12)	0.0186 (12)	0.0141 (11)	0.0042 (10)	0.0014 (9)	0.0044 (9)
C2	0.0147 (11)	0.0160 (11)	0.0165 (11)	0.0039 (9)	0.0032 (9)	0.0051 (9)
C3	0.0148 (11)	0.0158 (11)	0.0153 (11)	0.0026 (9)	0.0019 (9)	0.0041 (9)
C4	0.0136 (11)	0.0193 (11)	0.0141 (11)	0.0045 (9)	0.0028 (9)	0.0058 (9)
C5	0.0172 (12)	0.0165 (11)	0.0180 (11)	0.0049 (9)	0.0037 (9)	0.0068 (9)
C6	0.0127 (11)	0.0138 (11)	0.0197 (12)	0.0018 (9)	0.0030 (9)	0.0041 (9)
C7	0.0141 (11)	0.0188 (11)	0.0142 (11)	0.0036 (9)	0.0006 (9)	0.0042 (9)
C8	0.0187 (12)	0.0204 (12)	0.0145 (11)	0.0005 (9)	−0.0020 (9)	0.0033 (9)
C9	0.0176 (12)	0.0209 (12)	0.0143 (11)	−0.0016 (9)	−0.0031 (9)	0.0084 (9)
C10	0.0192 (12)	0.0163 (11)	0.0189 (12)	0.0007 (9)	−0.0046 (9)	0.0047 (9)
C11	0.023 (3)	0.018 (2)	0.017 (2)	0.005 (2)	−0.0006 (19)	0.002 (2)

### 2-Methyl-1,4-phenylene bis(3,5-dibromobenzoate) Geometric parameters (Å, º)

**Table:**

Br1—C4	1.896 (2)	C6—C7	1.381 (3)
Br2—C6	1.893 (2)	C7—H7	0.9500
O1—C1	1.199 (3)	C8—H8	0.9500
O2—C1	1.361 (3)	C8—C9	1.385 (3)
O2—C9	1.411 (3)	C8—C10^i^	1.395 (3)
C1—C2	1.493 (3)	C9—C10	1.381 (3)
C2—C3	1.397 (3)	C10—H10	0.94 (2)
C2—C7	1.393 (3)	C10—C11	1.504 (5)
C3—C4	1.380 (3)	C11—H10	0.56 (2)
C3—H3	0.945 (10)	C11—H11A	0.9800
C4—C5	1.387 (3)	C11—H11B	0.9800
C5—C6	1.389 (3)	C11—H11C	0.9800
C5—H5	0.951 (10)		
			
C1—O2—C9	117.81 (18)	C9—C8—H8	120.6
O1—C1—O2	124.3 (2)	C9—C8—C10^i^	118.7 (2)
O1—C1—C2	124.8 (2)	C10^i^—C8—H8	120.6
O2—C1—C2	110.85 (19)	C8—C9—O2	120.2 (2)
C3—C2—C1	117.2 (2)	C10—C9—O2	116.5 (2)
C7—C2—C1	122.0 (2)	C10—C9—C8	123.1 (2)
C7—C2—C3	120.7 (2)	C8^i^—C10—H10	124 (4)
C2—C3—H3	117.8 (18)	C8^i^—C10—C11	122.9 (3)
C4—C3—C2	118.6 (2)	C9—C10—C8^i^	118.2 (2)
C4—C3—H3	123.5 (18)	C9—C10—H10	118 (4)
C3—C4—Br1	119.26 (18)	C9—C10—C11	118.9 (3)
C3—C4—C5	122.0 (2)	C11—C10—H10	1 (4)
C5—C4—Br1	118.69 (17)	C10—C11—H10	1 (8)
C4—C5—C6	118.0 (2)	C10—C11—H11A	109.5
C4—C5—H5	120.9 (18)	C10—C11—H11B	109.5
C6—C5—H5	121.0 (18)	C10—C11—H11C	109.5
C5—C6—Br2	118.44 (17)	H10—C11—H11A	110.7
C7—C6—Br2	119.77 (18)	H10—C11—H11B	109.0
C7—C6—C5	121.8 (2)	H10—C11—H11C	108.8
C2—C7—H7	120.6	H11A—C11—H11B	109.5
C6—C7—C2	118.8 (2)	H11A—C11—H11C	109.5
C6—C7—H7	120.6	H11B—C11—H11C	109.5
			
Br1—C4—C5—C6	−177.72 (16)	C2—C3—C4—C5	0.5 (3)
Br2—C6—C7—C2	−177.37 (17)	C3—C2—C7—C6	−0.1 (3)
O1—C1—C2—C3	7.4 (3)	C3—C4—C5—C6	0.0 (3)
O1—C1—C2—C7	−170.4 (2)	C4—C5—C6—Br2	177.45 (17)
O2—C1—C2—C3	−173.95 (19)	C4—C5—C6—C7	−0.5 (3)
O2—C1—C2—C7	8.2 (3)	C5—C6—C7—C2	0.5 (3)
O2—C9—C10—C8^i^	176.3 (2)	C7—C2—C3—C4	−0.4 (3)
O2—C9—C10—C11	−2.3 (4)	C8—C9—C10—C8^i^	0.6 (4)
C1—O2—C9—C8	−65.1 (3)	C8—C9—C10—C11	−178.0 (3)
C1—O2—C9—C10	119.1 (2)	C9—O2—C1—O1	0.6 (3)
C1—C2—C3—C4	−178.3 (2)	C9—O2—C1—C2	−178.03 (19)
C1—C2—C7—C6	177.7 (2)	C10^i^—C8—C9—O2	−176.1 (2)
C2—C3—C4—Br1	178.15 (16)	C10^i^—C8—C9—C10	−0.6 (4)

Symmetry code: (i) −*x*+1, −*y*+2, −*z*.

### 2-Methyl-1,4-phenylene bis(3,5-dibromobenzoate) Hydrogen-bond geometry (Å, º)

**Table:**

*D*—H···*A*	*D*—H	H···*A*	*D*···*A*	*D*—H···*A*
C5—H5···Br1^ii^	0.95 (1)	3.12 (2)	3.893 (2)	139 (2)
C3—H3···Br1^iii^	0.95 (1)	3.02 (1)	3.956 (2)	172 (2)

Symmetry codes: (ii) −*x*+1, −*y*+1, −*z*+1; (iii) −*x*+2, −*y*+2, −*z*+1.
