# Supplementary figures and images for: Crystal and mol­ecular structure of 2-methyl-1,4-phenyl­ene bis­(3,5-di­bromo­benzoate)
Source: Acta Crystallogr E Crystallogr Commun. 2024 Jul 15;80(Pt 8):863–6. doi: 10.1107/S2056989024006820 (PMC11299744; doi:10.1107/S2056989024006820)

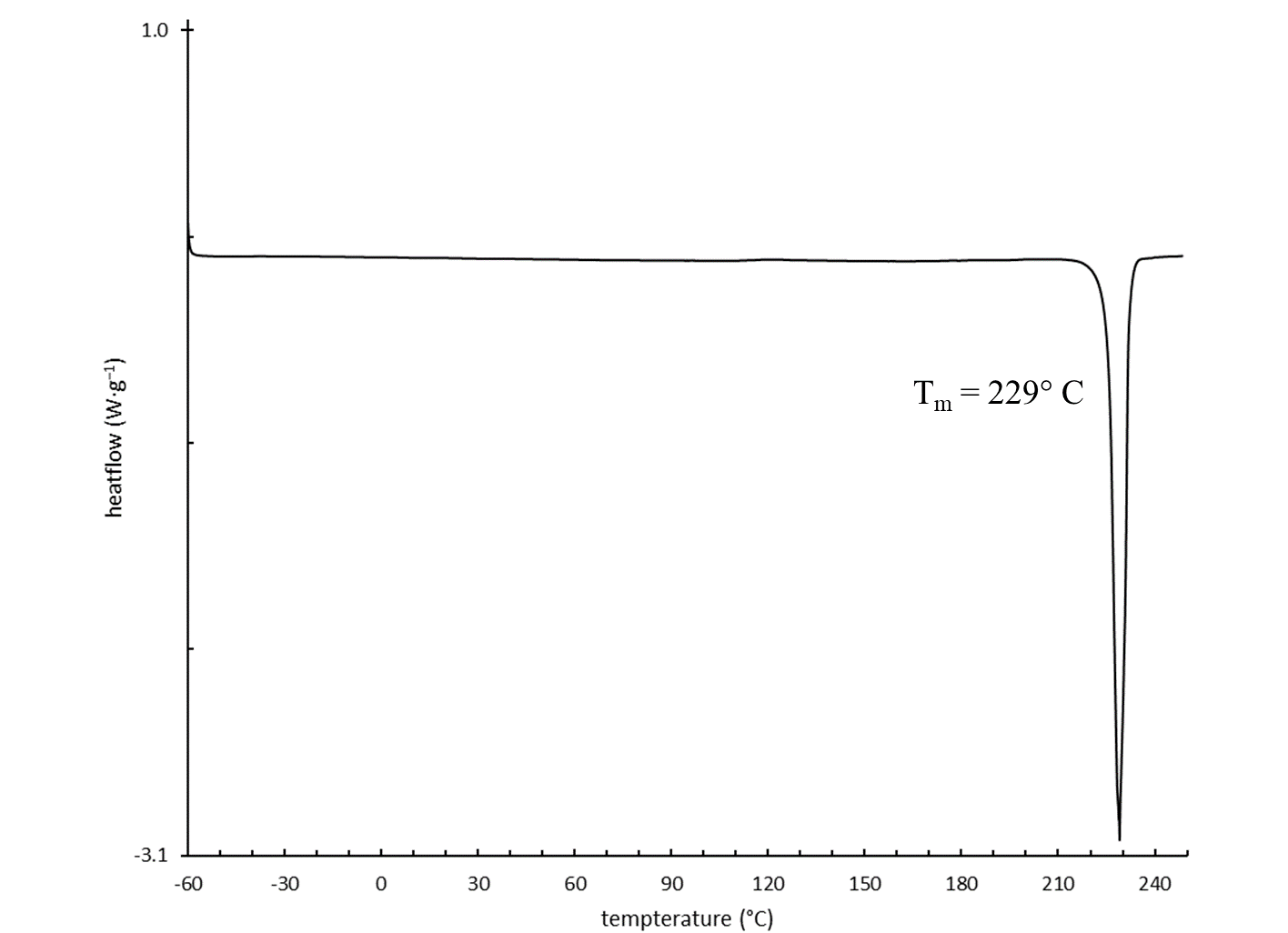

Supplement: Supplementary file 4 [file e-80-00863-sup4.tif]

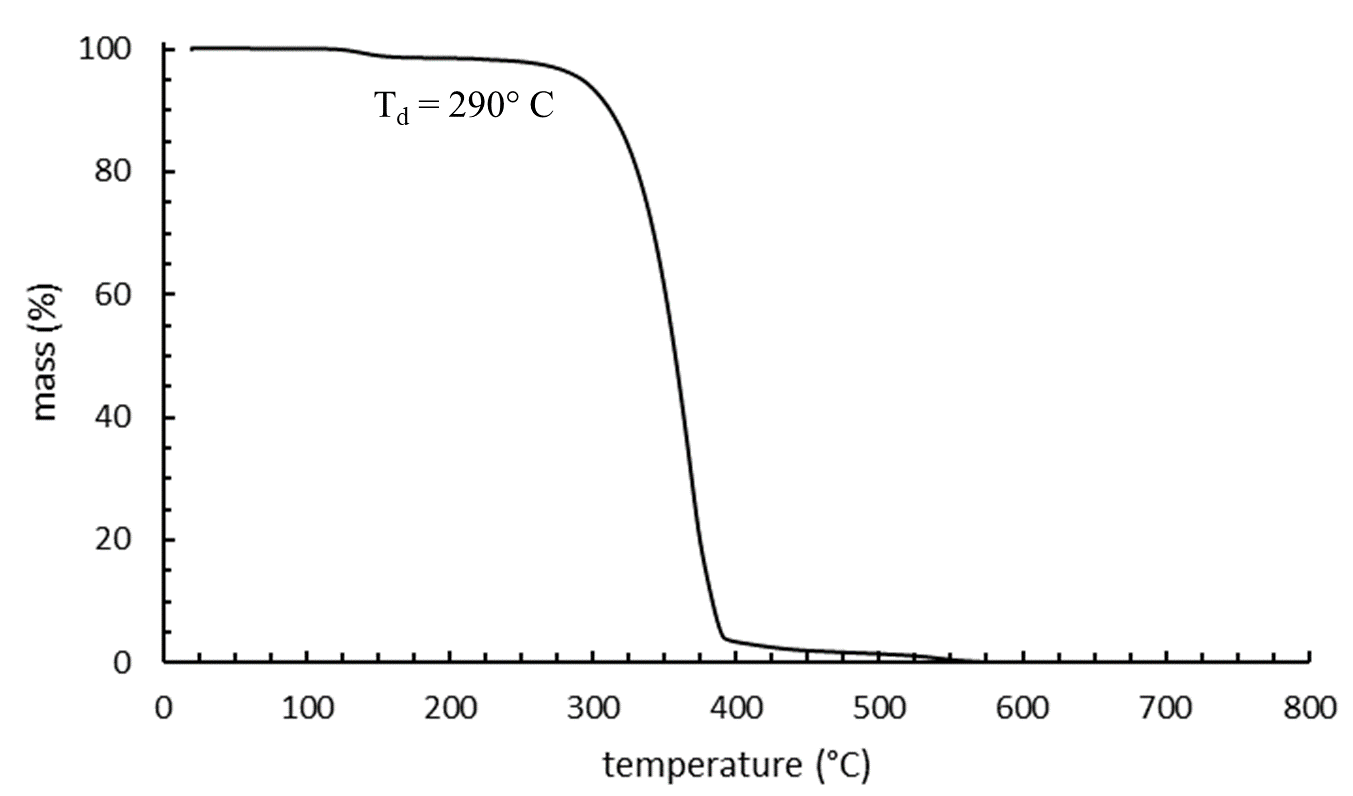

Supplement: Supplementary file 5 [file e-80-00863-sup5.tif]
